# Engineering of Shikimate Pathway and Terminal Branch for Efficient Production of L-Tryptophan in *Escherichia coli*

**DOI:** 10.3390/ijms241411866

**Published:** 2023-07-24

**Authors:** Shuai Liu, Bing-Bing Wang, Jian-Zhong Xu, Wei-Guo Zhang

**Affiliations:** The Key Laboratory of Industrial Biotechnology, Ministry of Education, School of Biotechnology, Jiangnan University, 1800# Lihu Road, Wuxi 214122, China

**Keywords:** *Escherichia coli*, L-tryptophan, metabolic engineering, fermentation optimization

## Abstract

L-tryptophan (L-trp), produced through bio-manufacturing, is widely used in the pharmaceutical and food industries. Based on the previously developed L-trp-producing strain, this study significantly improved the titer and yield of L-trp, through metabolic engineering of the shikimate pathway and the L-tryptophan branch. First, the rate-limiting steps in the shikimate pathway were investigated and deciphered, revealing that the combined overexpression of the genes *aroE* and *aroD* increased L-trp production. Then, L-trp synthesis was further enhanced at the shaking flask level by improving the intracellular availability of L-glutamine (L-gln) and L-serine (L-ser). In addition, the transport system and the competing pathway of L-trp were also modified, indicating that elimination of the gene *TnaB* contributed to the extracellular accumulation of L-trp. Through optimizing formulas, the robustness and production efficiency of engineered strains were enhanced at the level of the 30 L fermenter. After 42 h of fed-batch fermentation, the resultant strain produced 53.65 g/L of L-trp, with a yield of 0.238 g/g glucose. In this study, the high-efficiency L-trp-producing strains were created in order to establish a basis for further development of more strains for the production of other highly valuable aromatic compounds or their derivatives.

## 1. Introduction

**Figure 1:**
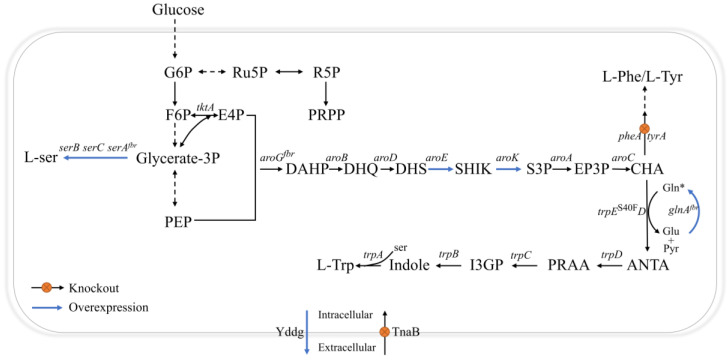
The metabolic engineering strategies employed in this study to enhance L-trp biosynthesis in *E. coli*. Overexpressed genes are indicated by blue arrows, blocked pathways are marked with orange crosses on black arrows, and dotted lines indicate that multi-step reactions are involved. *—acting as the donor of an amin group. Abbreviations: G6P—glucose-6-phosphate; Ru5P—ribulose-5-phosphate; R5P—ribose-5-phosphate; PRPP—phosphoribosyl-pyrophosphate; PEP—phosphoenolpyruvate; F6P—fructose-6-phosphate; Ser—serine; E4P—erythrose-4-phosphate; CHA—chorismate; DAHP—3-deoxy-D-arabino-heptulosonate-7-phosphate; Pyr—Pyruvate; PRAA—phosphoribosyl anthranilate; DHQ—3-Dehydroquinate; DHS—3-dehydroshikimate; SHIK—shikimate; EP3P—5-enolpyruvylshikimate 3-phosphate; ANTA—anthranilate; I3GP—indole 3-glycerolphosphate; S3P—shikimate-3-phosphate.

With continued advances in metabolic engineering and synthetic biology, the utilization of microbial cell factories to produce L-trp de novo has received a lot of attention, due to its sustainability and environmental friendliness (Table 1). As illustrated in Figure 1, the biosynthesis pathway of L-trp can be artificially divided into three modules: (1) the central metabolic pathway, which supplies the precursors phosphoenolpyruvate (PEP), erythrose-4-phosphate (E4P), 5-phospho-α-D-ribose 1-diphosphate (PRPP), L-ser, and L-gln (functioning as an amino donor); (2) the shikimate pathway, which connects the L-trp branch to the central metabolism; and (3) the L-trp branch. In the process of L-tryptophan biosynthesis, there are many negative regulatory mechanisms, such as feedback inhibition [1], feedforward regulation [2], repression [3], and attenuation [4], which exist in the shikimate pathway and L-tryptophan branch. In order to alleviate these negative regulatory mechanisms, strategies such as the overexpression of rate-limiting enzymes [5], the construction of anti-feedback variants [6], the blocking of degradation pathways [7], and the modification of transport systems [8] are often used. Despite this, the conversion rate of L-tryptophan is still far below the theoretical value (0.46 g/g glucose) [9], which is one of the key reasons for the high cost of L-tryptophan in industrial production. It is worth noting that, in the process of overexpressing pathway enzymes, researchers mostly focus on enhancing the expression of the L-tryptophan operon through promoter engineering or high-copy plasmids [2,10,11]. There are few studies on the possible rate-limiting sites in the shikimate pathway. In fact, in previous investigations, researchers discovered several intermediate metabolites from the shikimate pathway in the supernatant of the fermentation medium, which raised the possibility that the route may contain some restriction sites [12,13].

L-gln and L-ser are involved in the first and last steps of the L-tryptophan branch, and their intracellular concentrations are crucial for the biosynthesis of L-tryptophan. A previous study found that an inadequate supply of intracellular L-gln was an important variable limiting L-trp synthesis [14]. It is widely known that there is only one reaction in the L-gln biosynthetic pathway of *E. coli*. L-glutamate (L-glu) and ammonia are combined in this reaction via glutamine synthetase (encoded by *glnA*), resulting in L-gln. Recent research has shown that both extracellular and intracellular L-glu were accumulated during the fermentation process of L-trp-producing strains [1]. Actually, L-glu metabolism is very important for normal cell growth and the effective production of L-trp. On the one hand, ketoglutarate, the precursor to L-glu, is the connection point of carbon and nitrogen metabolism [18], and the intracellular contents of ketoglutarate and L-glu are two key signaling molecules for cells to adjust their own carbon and nitrogen balance [19]. On the other hand, the intracellular availability of L-gln is very important for L-trp biosynthesis [14]. Additionally, previous studies showed that, because of the weak enzyme kinetic properties of wild L-gln synthetase, it might not be possible to increase the availability of L-gln by improving the substrate L-glu, which is available in a nearly saturated range [1,20,21]. Therefore, maintaining intracellular L-gln at an appropriate concentration through metabolic engineering can help to balance the efficient synthesis of L-tryptophan and normal cell growth. In the final step of the L-trp branch, indole and L-ser are condensed to L-trp and catalyzed by L-trp synthase (encoded by *trpBA*) (Figure 1), which is generally considered to be another key site limiting the effective synthesis of L-trp. In order to remove the restriction, a feedback resistance variant, *serA*^H344A/N364A^ [2,5,6,14], and *serB*/*serC* [5] were overexpressed, resulting in the enhancement of intracellular L-ser concentration.

In order to avoid the accumulation of by-products L-phenylalanine (L-phe) and L-tyrosine (L-Tyr) in the medium, which would result in a decrease in the conversion rate of L-tryptophan relative to the substrate glucose and difficulty of the subsequent extraction and refining processes, previous studies adopted the strategy of directly knocking out the competitive branches of L-phe and L-Tyr [5,22]. However, L-phe and L-tyr are primary metabolites required for cell growth. Adding L-phe and L-tyr to the medium is necessary in order to restore normal cell growth after knocking out the competitive branch. It remains to be seen whether the benefits of eliminating this competitive branch can cover extra costs. L-tryptophan is usually believed to be transported to the extracellular space through simple diffusion, due to its hydrophobicity. However, some studies have suggested that modifying the transport system of L-tryptophan will help to decrease the concentration of intracellular L-tryptophan and weaken the negative regulation mechanism caused by a high concentration of intracellular L-tryptophan [8,23], though L-trp-specific transporters have not been reported so far.

Overall, based on a previously developed L-trp-producing strain stored in our laboratory, this study eliminated the rate-limiting sites in the shikimate pathway, improved the intracellular supply of L-gln and L-ser, and investigated the effects of the modification of the L-trp transport system and the elimination of competing routes on L-trp production. Finally, the production efficiency of the engineered L-trp strain was further improved through the optimization of medium composition at the level of the 30 L fermenter. After 42 h of fed-batch fermentation, the titer of engineered strain T13 reached 53.65 g/L, with a yield of 0.238 g/g glucose. The L-trp-producing strains produced in this study provide the basis for the further development of other aromatic compounds and their derivative-producing strains.

## 2. Results and Discussion

### 2.1. Identifying and Relieving Rate-Limiting Steps in the Shikimate Pathway

**Figure 2:**
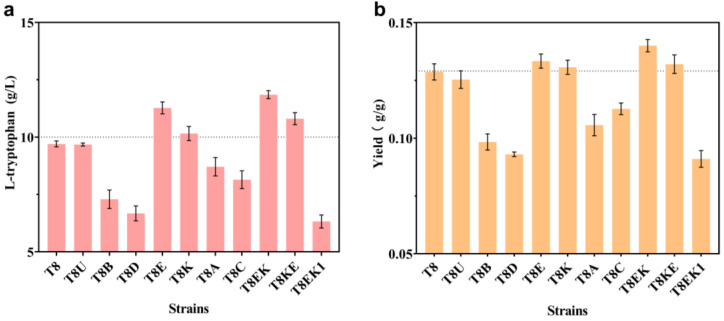
Identifying the rate-limiting steps in the shikimate pathway. (**a**) Effect of overexpression of the shikimate pathway genes on L-trp titer. (**b**) Effect of overexpression of the shikimate pathway genes on L-trp yield. All of the data illustrated in the graphs are presented as the mean and standard deviation and are from three parallel experiments.

As shown in supplementary Figure S1, a series of plasmids (pSB4K5-*aroB*, pSB4K5-*aroD*, pSB4K5-*aroE*, pSB4K5-*aroK*, pSB4K5-*aroA*, and pSB4K5-*aroC*) were created in this study in order to explore the potential rate-limiting steps in the shikimate pathway. These overexpressed genes (*aroB*, *aroD*, *aroE*, *aroK*, *aroA*, and *aroC*) were generated from the genome of *E. coli* W3110 and regulated via the *trc* promoter. During the procedure, the low-copy plasmid pSB4K5 was used as the framework. These resultant plasmids (including the original unloaded plasmid pSB4K5, pSB4K5-*aroB*, pSB4K5-*aroD*, pSB4K5-*aroE*, pSB4K5-*aroK*, pSB4K5-*aroA*, and pSB4K5-*aroC*) were sequentially introduced into strain T8 in order to generate strains T8U, T8B, T8D, T8E, T8K, T8A, and T8C (Table 2). As shown in Figure 2, when *aroE* and *aroK* were overexpressed (corresponding to strains T8E and T8K), the L-trp titer increased from 9.70 g/L to 11.27 g/L and 10.16 g/L, and the yield improved from 0.129 g/g to 0.133 g/g and 0.131 g/g, respectively. These results suggest that the insufficient expressions of *aroE* and *aroK* are really limiting factors for L-trp production. In a previous study, it was found that the intracellular concentrations of dehydroshikimic acid and shikimate in an engineered L-trp-producing strain increased rapidly in the middle stage of fermentation [1]. This implies that there may be one or several rate-limiting sites in the shikimate pathway that lead to the accumulation of intermediate metabolites. Next, in order to confirm the impact of the combined expressions of *aroE* and *aroK* on the production of L-trp and, meanwhile, avoid the titer fluctuations caused by plasmid instability [1,24], the relative locations of the genes *aroE* and *aroK* were adjusted on the artificial operon, and two artificial mini-operons (i.e., *aroEK* and *aroKE*), controlled by the *trc* promoter, were integrated into the pseudogene *ycjv* locus of T8, generating the T8EK and T8KE strains, respectively. The fermentation results showed that T8EK performed better at shake-flask level, with a 22% increase in titer and an 8.8% increase in yield, compared to T8 (i.e., 11.85 g/L vs. 9.70 g/L and 0.14 g/g vs. 0.13 g/g, respectively) (Figure 2). This recombinant strain (T8EK) was named T9. Based on strain T9, strain T8EK1 was created by inserting a second copy of *aroEK* into the pseudogene *mbhA* locus. Regrettably, both the titer and the yield of strain T8EK1 declined (Figure 2). It is speculated that further overexpression of shikimate pathway genes would cause a heavy burden on the normal physiological metabolism of cells [25]. In this study, based on strategies of plasmid overexpression, as well as the construction and integration of an artificial operon, we explored the rate-limiting steps in the shikimate pathway. In the past, in order to identify and remove impediments on the common pathway of aromatic amino acids, the analysis of culture supernatant samples, via NMR, was used [13]. In this study, using a straightforward gene overexpression strategy, without considering prior knowledge of pathways, rate-limiting sites were explored and resolved.

### 2.2. Enhancing Intracellular Availability of L-gln and L-ser

**Figure 3:**
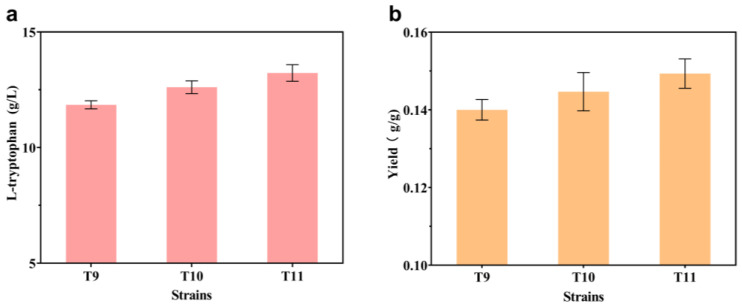
Impact of improved availability of L-gln and intracellular L-ser on L-trp biosynthesis. (**a**) L-trp titers of strains T9, T10, and T11 at shake flask level. (**b**) L-trp yields of strains T9, T10, and T11 at shake flask level. All of the data illustrated in the graphs are presented as the mean and standard deviation and are from three parallel experiments.

**Figure 4:**
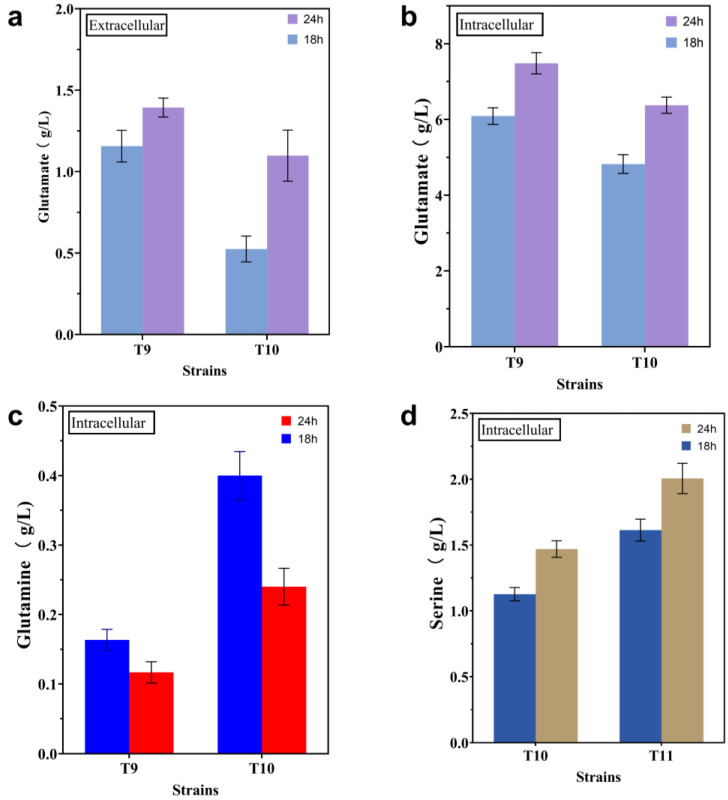
Amounts of extracellular and intracellular L-glu (**a**,**b**), intracellular L-gln (**c**), and intracellular L-ser (**d**) of strains T9, T10, and T11. All of the data illustrated in the graphs are presented as the mean and standard deviation and are from three parallel experiments.

The L-trp branch begins with the conversion of chorismate and L-gln to anthranilate, which is catalyzed by trpED. Equimolar quantities of L-glutamate and pyruvate are produced as a result of this reaction (Figure 1). Controlled by the *trc* promoter, a copy of the *glnA*^L159I, E304A^ variant, derived from *Bacillus subtilis* [20], was integrated into the pseudogene *mbhA* locus of T9 to produce strain T10. This strategy has been shown to increase the availability of intracellular L-gln [14]. The shake-flask results showed that, compared with strain T9, both the titer and the yield of strain T10 increased, from 11.85 g/L to 12.61 g/L and from 0.140 g/g to 0.145 g/g, respectively (Figure 3). In order to confirm that the insufficient amount of intracellular L-gln is indeed the limiting factor for the effective synthesis of L-trp, the levels of extracellular L-glu and L-gln in strains T9 and T10 were evaluated, using a quantitative approach. In comparison to strain T9 (Figure 4a), the extracellular L-glu content of strain T10 dropped from 1.16 g/L and 1.39 g/L to 0.53 g/L and 1.10 g/L at 18 h and 24 h, respectively. Meanwhile, the levels of intracellular L-glu reduced from 6.09 g/L and 7.48 g/L to 4.82 g/L and 6.38 g/L, respectively (Figure 4b). In contrast, the intracellular L-gln level increased from 0.16 g/L and 0.12 g/L to 0.40 g/L and 0.24 g/L at 18 h and 24 h, respectively (Figure 4c), but none was found in the extracellular medium. These results suggest that the introduction of the *glnA*^L159I, E304A^ variant promoted the conversion of intracellular L-glu to L-gln and contributed to the synthesis of L-trp. In order to further increase the availability of intracellular L-gln, another copy of the *glnA*^L159I, E304A^ variant was incorporated into the genome of strain T10, but the L-trp titer did not increase.

L-ser is an important precursor for the synthesis of L-trp, and improving intracellular L-ser availability through overexpression of feedback-resistant *serA*^H344A,N364A^ is a common engineering strategy [6]. At the same time, it is also noted that an excessive intracellular L-ser concentration will hinder the normal growth of *E. coli* [27], while inadequate L-ser levels will hamper the efficient production of L-trp [14]. Therefore, increasing the availability of intracellular L-ser, while avoiding cytotoxicity, will theoretically facilitate the development of engineered strains with higher yields. In order to further promote the intracellular L-ser supply, one copy of *serB* and *serC,* regulated by their original promoters, was introduced into the pseudogene *yeeP* locus of strain T10, generating strain T11. The results showed that the L-trp titer and yield of strain T11 increased to 13.23 g/L and 0.149 g/g, respectively (Figure 3). In order to examine if the increase in L-trp production is actually due to the overexpression of the *serB* and *serC* genes, the extracellular and intracellular L-ser concentrations of strains T10 and T11 were detected. The results showed that the intracellular L-ser level increased from 1.13 g/L and 1.47 g/L to 1.61 g/L and 2.01 g/L at 18 h and 24 h, respectively (Figure 4d), while no L-ser was found in the medium. This demonstrates that the production capacity of the engineered strain T10 is indeed constrained by the insufficiency of L-ser, and increased expressions of the genes *serB* and *serC* help with L-trp biosynthesis. Another copy of *serB* and *serC* was integrated into the genome of strain T11, but no better results were obtained. A recent study found that overexpression of serC has a favorable influence on L-trp production, based on metabolic control analyses from short-term perturbation trials [25]. This study’s conclusion is in line with the findings of our experiment.

### 2.3. Modification of the L-trp Transport System

**Figure 5:**
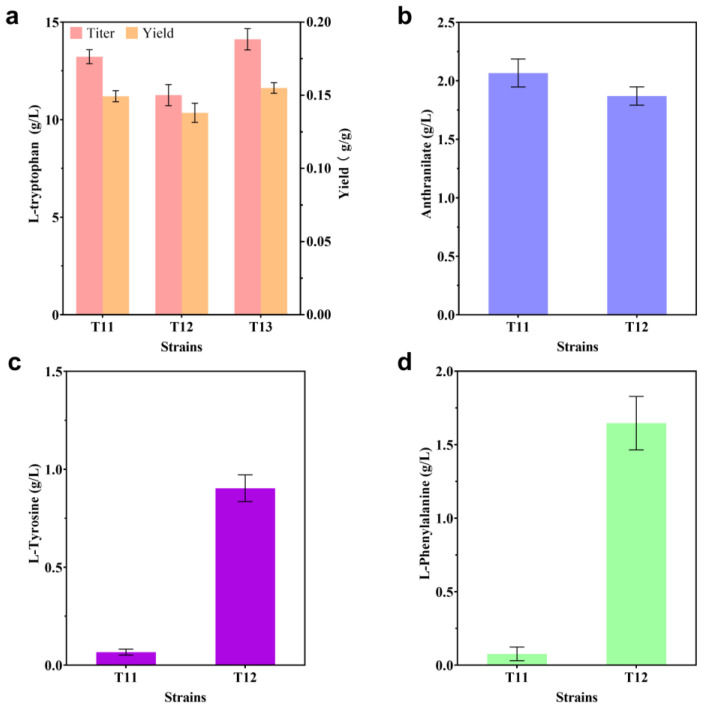
Influence of modification of the L-trp transport system via overexpression of the gene *yddG* or deletion of the gene *TnaB* on the production of L-trp (**a**), anthranilate intracellularly (**b**), L-tyrosine extracellularly (**c**), and L-phenylalanine extracellularly (**d**). All of the data illustrated in the graphs are presented as the mean and standard deviation and are from three parallel experiments.

Modifying the transport system of the desired chemical is just as important as rewiring the synthesis route and regulatory network in building an effective microbial cell factory, and this strategy is widely used to build hyperproducing strains for bulk amino acid products, such as lysine [28]. According to earlier studies, the *yddG* gene in *E. coli* encodes an inner membrane protein, which belongs to the drug/metabolite transporter (DMT) superfamily and is responsible for the extracellular transport of aromatic amino acids [29,30]. *yddG* overexpression was used in previous research to create L-trp-producing bacteria [5,8]. In this study, a variety of strains based on T11 were produced through in situ *yddG* overexpression. Unexpectedly, no matter how the promoter intensity was altered, the L-trp titer did not increase. Interestingly, when *yddG* was placed under the control of the *trc* promoter (named T12), white crystals appeared on the wall of the shake flask after fermentation. Initially, this phenomenon was considered to be caused by precipitation as a result of the low solubility of L-trp. However, after adding water to dissolve the crystals and measuring again, it was found that the titer of L-trp did not increase (Figure 5a). In order to understand the reason behind this result, we measured the intracellular and extracellular contents of anthranilate in T11 and T12 strains, and it was found that there was no accumulation of anthranilate outside the cells of T11 and T12. However, as compared to 2.07 g/L of T11, the intracellular anthranilate concentration of T12 fell to 1.87 g/L (Figure 5b), which was consistent with prior work [5]. It was hypothesized that the overexpression of *yddG* under the control of the *trc* promoter promoted the extracellular translocation of L-tyr and L-phe. In order to test this hypothesis, the concentrations of L-tyr and L-phe in the extracellular media of strains T11 and T12 were measured. It was found that only very small amounts of L-tyr and L-phe accumulated extracellularly for strain T11, whereas the accumulations for strain T12 reached 0.90 g/L and 1.65 g/L, respectively (Figure 5c,d). These experimental results show that overexpression of *yddG* can significantly stimulate the extracellular transport of L-tyr and L-phe. A decrease in intracellular anthranilate content may be caused by an increase in carbon flow into the L-phe and L-tyr branches. Indeed, specific L-trp carriers have not been identified for many years. Transcriptomic mining [31], sequence similarity searching [32], and protein engineering [33] may be among the future feasible solutions for obtaining L-trp-specific transporters.

The *tnaB* gene encodes an aromatic amino acid permease, which is the primary carrier for L-trp uptake [23,34]. The T13 strain was created by knocking out the gene *tnaB* from strain T11 to prevent fruitless cycles. The results showed that the L-trp titer in strain T13 increased to 14.12 g/L, and the yield reached 0.155 g/g (Figure 5a), which were 6.7% and 4.0% higher, respectively, than those of strain T11. These results are in agreement with a previous study [23]. Knocking out *tnaB* prevents extracellular L-trp from reentering the cell and helps mitigate the impact of a high intracellular concentration of L-trp on a normal physiological metabolism.

### 2.4. Blocking Competing Pathways

**Figure 6:**
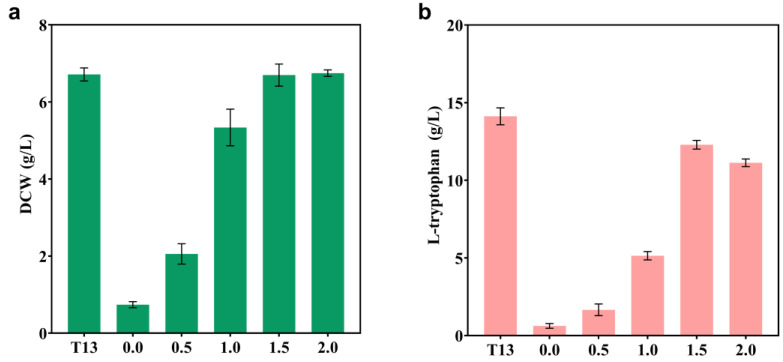
The impact of adding L-phe and L-tyr in varying doses (0–2 g/L) on the biomass and L-trp titer of strain T14. (**a**) Compared with the strain T13, the DCW of T14 at different concentrations of L-phe and L-tyr at the shake flask level. (**b**) Compared with the strain T13, the L-trp titer of T14 at different concentrations of L-phe and L-tyr at the shake flask level. All of the data presented in the graphs are illustrated as the mean and standard deviation and are from three parallel experiments.

Removing competing pathways in order to redirect more carbon flow into the synthesis pathway of target chemicals is a common engineering strategy [35,36,37]. As shown in Figure 1, the metabolite chorismate, at the end of the shikimate pathway, can be further converted into L-phe, L-tyr, and L-trp. In order to avoid wasteful carbon flow, a recent study adopted the strategy of knocking out the genes *pheA* and *tyrA,* to enhance the biosynthesis of L-trp [5]. Based on strain T13, the auxotrophic strain T14 for L-phe and L-Tyr was developed using the same methodology. First, L-phe and L-tyr were added in varying concentrations in order to restore the cell growth. The highest biomass of the T14 strain was only 0.74 g/L (Figure 6), with no additional L-phe or L-tyr. As both nutrients were added, both the biomass and the titer of L-trp gradually increased. With the addition of a minimum of 1.5 g/L of L-phe and L-tyr individually, the T14 strain resumed normal growth (Figure 6a). However, the titer of strain T14 did not increase as much as expected, and instead decreased compared to strain T13. When 1.5 g/L or 2 g/L of L-phe and L-tyr were added, the L-trp titers of T14 were 12.29 g/L and 11.13 g/L, respectively, which were lower than that of strain T13 (14.12 g/L) (Figure 6b). This phenomenon can be explained by the fact that the terminal branches of L-phe and L-tyr are subject to a strict regulation mechanism, and it is unnecessary to interrupt the L-phe and L-tyr terminal pathways [38]. Additionally, the additional L-phe and L-tyr will increase the cost of manufacturing.

### 2.5. Optimization of L-trp Production in a 30 L Triple Fermenter

**Figure 7:**
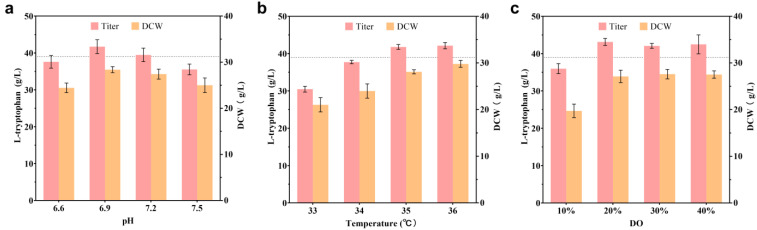
The impact of single-factor optimization (pH, temperature, and dissolved oxygen) on the L-trp titer and biomass at the 30 L fermenter level. (**a**) The pH levels are set at 6.6, 6.9, 7.2, and 7.5, the temperature at 36 °C, and the DO at 40%. (**b**) The temperature levels are set at 33, 34, 35, and 36 °C, the pH at 6.9, and the DO at 40%. (**c**) The DO levels are set at 10%, 20%, 30%, and 40%, the pH at 6.9, and the temperature at 35 °C. All of the data illustrated in the graphs are presented as the mean and standard deviation and are from three parallel experiments.

Optimizing culture conditions is crucial for achieving the best yield of engineered bacteria. First, in a triple fermenter of 30 L in fed-batch mode, the fermentation conditions for strain T13, which included the pH, culture temperature, and dissolved oxygen level, were optimized at the single-factor level. Results showed that the best conditions for strain T13 were 35 °C, pH 6.9, and DO 20% (Figure 7).

**Figure 8:**
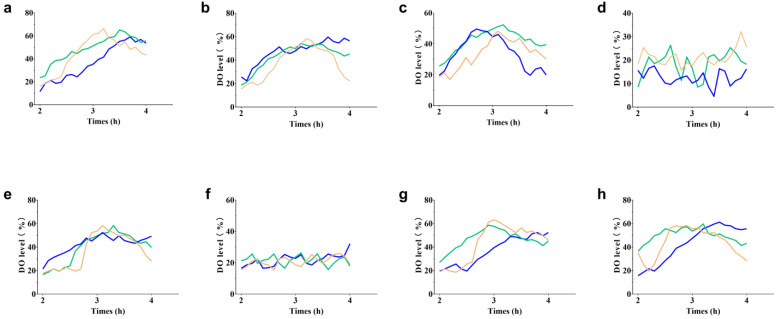
Effects of various nutrient additions on the fluctuation of DO in T13 during the early stages of fermentation. (**a**) Original medium; (**b**) 2 g/L of peptone; (**c**) 2 g/L of yeast extract; (**d**) 20 g/L of corn steep liquor; (**e**) 0.5 g/L of L-leucine; (**f**) 0.5 g/L of L-isoleucine; (**g**) 0.5 g/L of L-alanine; and (**h**) 0.5 g/L of L-proline. All of the data shown in the graphic come from three parallel experiments and curves in three colors represent data from three fermenters in parallel.

**Figure 9:**
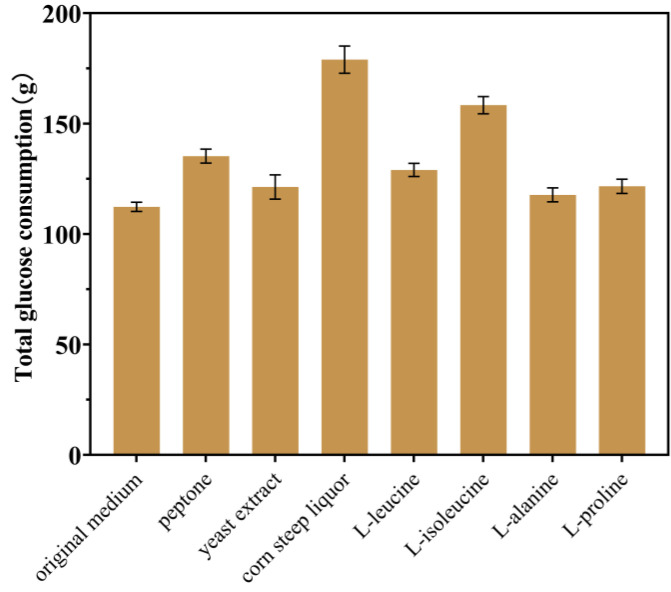
Effects of different nutrient additions on the glucose consumption of strain T13 during the early stages (2–4 h) of fermentation. The final concentrations of peptone and yeast extract are 2 g/L and 4 g/L, respectively. The final concentration of corn steep liquor is 20 g/L, and the final concentrations of L-leucine, L-isoleucine, L-alanine, and L-proline are all 0.5 g/L. All of the data illustrated in the graph are presented as the mean and standard deviation and are from three parallel experiments.

**Figure 10:**
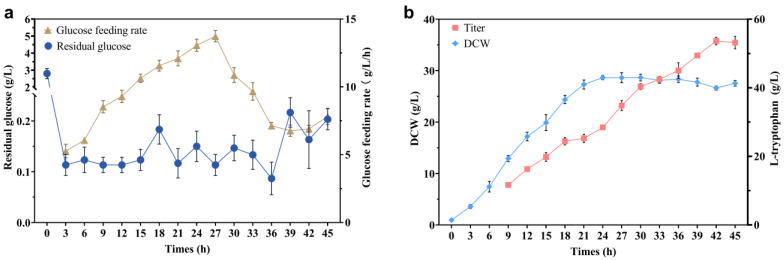
Fed-batch production of L-trp in a 30 L triple fermenter using strain T13. The fermentation medium was supplemented with 0.5 g/L of L-ile. (**a**) The feeding rate and glucose concentration during the fermentation process. (**b**) L-trp and biomass accumulation with fermentation time. Every three hours, samples were taken for measurement and calculation. Biomass was converted into dry cell weight (DCW) for measurement. All of the data illustrated in the graphs are presented as the mean and standard deviation and are from three parallel experiments.

Based on the single-factor optimization results, the fermentation performance of strain T13 was evaluated in a triple 30-L tank. During this process, when the original glucose of 10 g/L was exhausted, 600 g/L of sterilized glucose was immediately added, and the glucose concentration in the medium was kept at about 0.1 g/L. Unfortunately, in almost all experimental fermentation batches (excluding batches for which the stirring speed or ventilation rate was artificially reduced in advance), the dissolved oxygen in the early stage (2–4 h) of fermentation increases abnormally (Figure 8), which is unusual. Because this abnormal phenomenon occurs in the early stages of fermentation, we initially thought that it was caused by insufficient nutrition. The conventional organic nitrogen sources peptone, yeast extract powder, and corn steep liquor were added to the fermentation medium; as shown in Figure 8b–d, only when 20 g/L corn steep liquor was added to the culture medium (Figure 8d) did the anomalous increase in DO disappear. In addition, adding 20 g/L of corn steep liquor can greatly increase glucose absorption (Figure 9). The total glucose consumption between 2 and 4 h increased by 50.4% when 20 g/L of corn steep liquor was supplied, compared to the control group (original medium). But DO suddenly increased after 6–8 h, accompanied by cell growth stagnation. The sample of corn steep liquor is believed to contain active ingredients that can help solve the problem of abnormally elevated DO. Meanwhile, cell growth was increasingly hindered by various low-level hazardous chemicals in the corn steep liquor, including sulfite, with prolonged culture times. In order to identify the active ingredients, the concentrations of various amino acids in the corn steep liquor were detected using HPLC, in which L-leucine, L-isoleucine, L-alanine, and L-proline were found to be relatively abundant (Table 3). These four amino acids were separately added to the fermentation medium for verification (Figure 8e–h). It was found that, when 0.5 g/L of isoleucine was added, the abnormally increased DO disappeared (Figure 8f), and the glucose consumption increased by 41.2% compared to that of the control group (Figure 9). The absence of the essential amino acid for cell growth may be the cause of abnormal DO in the early stages of fermentation. A previous study showed that the synthesis of L-ile was inhibited by the excessive accumulation of L-ser in *E. coli* [27,39]. In order to confirm this hypothesis, the genes *serB* and *serC* in strain T13, which were previously introduced at the *yeeP* site, were eliminated to create strain T15. The results showed that the abnormal DO increase disappeared, but the titer of strain T15 decreased significantly (12.71 g/L). Finally, 0.5 g/L of L-isoleucine was added to the fermentation medium, and strain T13 produced 53.65 g/L of L-trp and reached a yield of 0.238 g/g after 42 h of culture through fed-batch fermentation (Figure 10). Therefore, it can be concluded that the excessive or inadequate availability of intracellular L-ser adversely affects the effective production of L-trp, and a slight oversupply of intracellular serine contributes to the high production of L-trp. Of course, the precise control over the intracellular concentration of L-ser by means of a dynamic control strategy will help to achieve a better realization of this idea [40].

## 3. Materials and Methods

### 3.1. Strains and Plasmids

*E. coli* jm109 was used for plasmid construction and propagation. Table 2 displays the genetic information of *E. coli* T8, which was used as the starting strain for the further improvement of L-trp synthesis. The T8 strain was developed in a separate study conducted in our laboratory. In order to build T8, *E. coli* W3110, which underwent several rounds of mutagenesis and screening, was used as the starting strain, and *lacI* was first knocked out in order to facilitate the use of the *trc* promoter. P_trc_-*aroG*^S180F^, P_trc_-*trpE*^S40F^, and P_trc_-*serA*^H344A, N364A^ were then inserted into the *tyrR*, *trpE*, and *trpR* sites, respectively, in order to enhance the metabolic flow of the shikimate pathway and the L-trp branch. Moreover, the precursor phosphoenolpyruvate (PEP) and erythrose-4-phosphate (E4P) supply networks of this obtained recombinant strain were reprogrammed. The specific strategy included overexpressing *tktA* (encoding transketolase I) and deleting *pykf* (encoding pyruvate kinase I), followed by replacing the endogenous phosphoenolpyruvate:carbohydrate phosphotransferase system (PTS) with the PEP-independent galactose permease/glucokinase (GalP/Glk) system. In addition, the enhancement of glucose uptake was achieved by optimizing the expression levels of GalP and Glk at both the transcriptional and translational levels. Finally, with the help of the growth-stage-dependent promoters, the pyruvate generated in the L-trp branch was rationally redirected to the shikimate pathway in order to maximize the use of carbon atoms. The Crispr editing system, consisting of plasmid pCas9 and pTarget [26], was purchased from Addgene. The low-copy plasmid pSB4k5 was used to overexpress shikimate pathway genes, and its map is attached to Supplementary Figure S1. The overexpression plasmids (i.e., pSB4K5-*aroB*, pSB4K5-*aroD*, pSB4K5-*aroE*, pSB4K5-*aroK*, pSB4K5-*aroA*, and pSB4K5-*aroC*) used in this study were constructed using the commercial kit ClonExpress^®^ Ultra One Step Cloning Kit C115 (Vazyme, Nanjing, China), and the specific operating method referred to in its manual. All primers used in this study are listed in Table 4.

### 3.2. Gene Editing Using CRISPR/Cas9

A CRISPR-Cas9 system was used to implement gene integration or deletion [26]. The construction procedure utilized for the ptarget plasmids was the whole-plasmid PCR method. In detail, specific primers (designed through the online website Crispor, http://crispor.tefor.net/, accessed on 6 March 2021), containing the appropriate N20 sequences of target cassettes at the 5′ end were used to amplify the entire pTarget plasmid. The template plasmid was digested with DpnI at 37 °C for 45 min before the PCR products were transformed into *E. coli* jm109. Afterwards, individual colonies were selected and shaken, and then plasmids were extracted. The plasmid was used for subsequent experiments after the sequencing was correct. The donor fragment was obtained using overlapping PCR, with the upper and lower homology arms being set at about 500 bp in length. The donor fragment, which was used to eliminate the target gene, was created by combining the sequences of the upstream and downstream regions of the target gene. The upstream homologous arm, the target gene to be introduced, and the downstream homologous arm were fused to create donor fragments in order to integrate the genes into the genome. A gel extraction kit (DC301, Vazyme, Nanjing, China) was used to purify and recover the donor fragments.

For recombination, the pCas9 plasmid, containing red recombinase and Cas9 nuclease, was first introduced into the competent cells via chemical transformation. *E. coli* containing pCas9 plasmids was inoculated in the antibiotic-free LB medium and grown at 31 °C and 150 rpm until OD_600_ reached about 0.15. The expression of red recombinase was induced by adding arabinose with a final concentration of 10 mM. When OD_600_ reached 0.6, the cells were harvested.

For electroporated transformation, the pre-chilled 50 uL competent cells, 100 ng ptarget plasmids, and 500 ng purified donor fragments were mixed in an EP tube and immediately transferred to a 0.1 cm cuvette. Then, The Ptarget plasmids and donor fragments were transferred into competent cells using a Bio-Rad MicroPulser at 1.8 kV and 5.0 ms. The transformants were transferred to a shake tube containing 1 mL of LB medium and recovered at 31 °C and 150 rpm for 90 min after electroporation. The culture was then placed on a plate that contained 50 ug/mL kanamycin and 50 ug/mL streptomycin after centrifugation. After overnight culturing at 31 °C, single colonies were randomly selected, and positive transformants were identified using colony PCR and DNA sequencing. The shake tube medium containing positive transformants was then mixed with 0.1 mM IPTG at 31 °C and 150 rpm for 12 h to eliminate the ptarget plasmids. The pCas9 plasmids were removed via continuous culture at 42 °C.

### 3.3. Shake-Flask Fermentation

The target recombinant bacteria were scraped from the plate and transferred into a shake flask containing 30 mL of seed medium (containing 30 g/L glucose, 3 g/L yeast extract powder, 4 g/L (NH_4_)_2_SO_4_, 2 g/L sodium citrate, 1 g/L K_2_HPO_4_, 1 g/L KH_2_PO_4_, 10 mg/L FeSO_4_·7H_2_O, 10 mg/L MnSO_4_·H_2_O, 5 ug/L VB_1_, and 2 ug/L VH; pH 7.0) and cultured at 37 °C and 200 rpm for 8 h. In total, 3 mL of the seed cultures obtained were transferred into a shake flask that contained 27 mL of fermentation medium (containing 20 g/L glucose, 2 g/L sodium citrate, 7 g/L KH_2_PO_4_, 2 g/L yeast extract powder, 6 g/L (NH_4_)_2_SO_4_, 50 mg/L FeSO_4_·7H_2_O, 30 mg/L MnSO_4_·H_2_O, 2 ug/L VB_1_, and 1 ug/L VH; pH 7.0) and continued to be cultured for 24 h at 37 °C and 200 rpm. During the fermentation process, a syringe was used to add NH_4_OH (25%, *v*/*v*) and 60% glucose. A phenol red indicator and pH precision test paper were used to adjust the medium pH.

### 3.4. Fed-Batch Fermentation in a 30 L Bioreactor

A total of 30 mL of overnight seed culture was transferred to a 15-L bioreactor that contained 9 L of seed medium (containing 3 g/L yeast extract powder, 4 g/L (NH_4_)_2_SO_4_, 30 g/L glucose, 2 g/L sodium citrate, 2 g/L K_2_HPO_4_, 2 g/L KH_2_PO_4_, 30 mg/L FeSO_4_·7H_2_O, 20 mg/L MnSO_4_·H_2_O, 5 ug/L VB_1_, and 2 ug/L VH; pH 7.0) and cultivated at 36 °C, dissolved oxygen levels (DO) > 20%, and pH 7.0 until the OD_600_ reached 15–18. Thereafter, 2 L of seed culture was transferred to a 30-L bioreactor that contained 12 L of fermentation medium (containing 10 g/L glucose, 3 g/L sodium citrate, 7 g/L KH_2_PO_4_, 2 g/L yeast extract powder, 6 g/L (NH_4_)_2_SO_4_, 50 mg/L FeSO_4_·7H_2_O, 30 mg/L MnSO_4_·H_2_O, 3 ug/L VB_1_, and 1 ug/L VH; pH 6.9), and cultivation continued for 45 h. The pH during the fermentation process was controlled by feeding 25% ammonia-containing water. The DO level was controlled by adjusting the stirring speed, ventilation rate, feed rate, and air pressure in the bioreactor. The sterile glucose solution (600 g/L) was provided immediately after the initial glucose in the medium was exhausted. Fed-batch fermentations were carried out in three parallel trial tanks.

### 3.5. Analytical Methods

A spectrophotometer (UV-2600i, Shimadzu, Kyoto, Japan) was used to perform the OD_600_ measurements. The dry cell weight (DCW) was calculated using the following formula: DCW = OD_600_ multiplied by 0.36 (calibrated in this study). The glucose and L-glu concentrations during fermentation were measured using the SBA-40C biosensor (Shandong Province Academy of Sciences, China) as previously described [2]. A high-performance liquid chromatograph (Agilent 1200, Santa Clara, CA, USA), equipped with a Diamonsil C18 column, was used to detect the concentration of free amino acids in corn steep liquor. Specifically, 2,4-dinitrofluorobenzene (DNFB) was used to derivatize amino acids. The ratio between mobile phase A (sodium acetate solution with a pH of 6.5) the mobile phase B (methyl cyanide: water = 1:1) was set at 1:1. The flow rate was set at 1 mL/min, the detecting wavelength was set at 360 nm, and the temperature was set at 35 °C. The extracellular concentrations of L-trp, L-tyr, L-phe, and anthranilate were determined using previous methods [22]. Specifically, methanol and 0.05% H_3_PO_4_ were used as mobile phases, and an Inertsil ODS-SP column (250 mm × 4.6 mm i.d., 5 um, GL Sciences, Osaka, Japan) was used for gradient elution. A gradient elution procedure was used, and the detailed steps are presented in Table S1. For the L-trp assay, the sample had to be boiled for 10 min after the titer of L-trp was above 20 g/L. An equivalent volume of distilled water was added after it reached more than 45 g/L. The amounts (A_i_) of the intracellular metabolites L-glu, L-ser, and anthranilate were calculated using the following equation: A_i_ = T/V. T (g) refers to the total absolute number of intracellular metabolites, and V (liter) refers to the estimated intracellular volume. V can be obtained by multiplying the dry cell weight by the coefficient 0.0023 L/g (aqueous volume to cellular dry weight) for *E. coli* [41].

## 4. Conclusions

Based on a previously constructed L-trp-producing *E. coli* strain, this work found that the rate-limiting steps in the shikimate pathway were relieved by the combined overexpression of *aroE* and *aroK*. Improved intracellular availability of L-gln and L-ser contributed to higher L-trp production. Overexpression of *yddG* and blocking of competing pathways could not promote the production of L-trp, while knockout of the *tnaB* gene contributed to the accumulation of extracellular L-trp. The fermentation formula was optimized at the 30 L fermenter level in order to solve the problem of abnormal DO in the early stage of fermentation. After 42 h of fed-batch fermentation, the final L-trp titer reached 53.65 g/L, and the yield reached 0.238 g/g glucose. The research idea in this study provided a reference for constructing efficient cell factories for other aromatic compounds.

## Figures and Tables

**Table 1:** The previous studies on L-trp production in *E. coli*.

Strains	Carbon Source	Culturing Methods	Titer (g/L)	Yield (g/g)	Reference
SX11	glucose	5 L bioreactor Fed-batch	41.7	0.227	[6]
TRP12	glucose	5 L bioreactor Fed-batch	52.1	0.171	[5]
TS-10	glucose	shake-flask	1.71	NS	[14]
S028AA	glucose	1.5 L bioreactor Fed-batch	48.3	0.185	[15]
W3650/trpA-EBR/tnaA	glucose	100 L bioreactor Fed-batch *	>45 **	0.224	[16]
Dmtr/pta-Y	glucose	30 L bioreactor Fed-batch	52.6	0.202	[17]

*—anthranilic acid was fed. **—the data are estimated from the charts, since there is no clear data in the original text. NS—unavailable.

**Table 2:** Strains and plasmids used in this study.

Strains/Plasmids	Characteristics	Source
**Strains**		
*E. coli jm109*	the cloning host	This lab
T8	W3110, Δ*lacI*, *tyrR*::P_trc_-*aroG*^S180F^, *trpR*::P_trc_-*serA*^H344A,N364A^, *trpE*::P_trc_-*trpE*^S40F^, Δ*pykF*, *tktA*::P_trc_-*tktA*, Δ*ptsH*, *yjiV*::P_J23105_-UTR_WP1_-*galP*, *ylbE*::P_J23114_-UTR_MK3_-*glk*, PrpsT p1-*ppc*, Prrnc p1-*gltA*	This lab
T8U	T8, pSB4K5	This study
T8B	T8, pSB4K5-*aroB*	This study
T8D	T8, pSB4K5-*aroD*	This study
T8E	T8, pSB4K5-*aroE*	This study
T8K	T8, pSB4K5-*aroK*	This study
T8A	T8, pSB4K5-*aroA*	This study
T8C	T8, pSB4K5-*aroC*	This study
T8KE	*ycjv*::P_trc_-*aroKE*	This study
T9 (T8EK)	*ycjv*::P_trc_-*aroEK*	This study
T10	T9, *mbhA*::P_trc_-*glnA*^L159I,E304A^	This study
T11	T10, *yeeP*::P_serB_-*serB*, P_serC_-*serC*	This study
T12	T11, *yddG*::P_trc_- *yddG*	This study
T13	T11, Δ*TnaB*	This study
T14	T13, Δ*pheA*, Δ*tyrA*	This study
T15	T13, Δ*yeeP*::P_serB_-*serB*, P_serC_-*serC*	This study
**Plasmids**		
pCas9	repA101(Ts), P_cas_-*cas9*, kan, *lacI*^q^, P_araB_-Red, P_trc_-sgRNA-pMB1	[26]
pTarget	pMB1, *aadA*, sgRNA	[26]

**Table 3:** The quantities of free amino acids in the corn steep liquor used in this study.

Name	Content (mg/g)	Name	Content (mg/g)
Leucine	9.25 ± 0.21	Tryptophan	0.58 ± 0.19
Isoleucine	13.98 ± 0.31	Phenylalanine	7.12 ± 0.54
arginine	8.69 ± 0.18	Glutamate	6.35 ± 0.28
Alanine	12.56 ± 0.41	Lysine	6.85 ± 0.51
Histidine	3.92 ± 0.61	serine	3.32 ± 0.32
Glycine	1.36 ± 0.29	Valine	7.25 ± 0.41
Methionine	1.25 ± 0.51	Aspartate	3.96 ± 0.12
Asparagine	4.32 ± 0.31	proline	8.91 ± 0.19
threonine	4.58 ± 0.51		

All data are presented as the mean values of three measurements from three separate trials, plus standard deviation.

**Table 4:** Primers used in this study.

Name	Sequence
AroE-1	ctagctcccagtagacgaagttcgcgcac
AroE-2	gttcctcctactggatggcctgattcacg
AroE-3	cgtctactgggagctagagctgtcagacca
AroE-4	ccatccagtaggaggaactacactgtctgcag
aroK-1	cagagagcggagctagagctgtcagacc
aroK-2	ggtacagtaaggaggaactacactgtctgcag
aroK-3	ctagctccgctctctgagcgaagcg
aroK-4	agttcctccttactgtacccgcagacgagtg
N20-mbhA-F	gccaaaacctgccaccgtgggttttagagctagaaatagcaag
N20-mbhA-R	ccacggtggcaggttttggcactagtattatacctaggactgag
trc-glnA-F	ttgacaattaatcatccggctcgtataatgtgtggaaagaggaggaattttaccaaatgg
trc-glnA-R	ctgacacttttagtgccaagg
serBC-1	atgcggtataacgatgtcagcagccagc
serBC-2	gacatcgttataccgcatcaggcgct
serBC-3	tccattgccctgtcgtgactgatgccc
serBC-4	cgacagggcaatggatacaaggtagcctcatg
trcY-F	cctcaatagcggtagaattgacaattaatcatccggctcgtataatg
trcY-R	ggatgattaattgtcaattctaccgctattgaggtaggtcaattt
N20-tnaB-F	gccggtgcctggtttttctggttttagagctagaaatagcaag
N20-tnaB-R	cagaaaaaccaggcaccggcactagtattatacctaggactgag

## Data Availability

The data presented in this study are available upon request from the corresponding author.

## Supplementary Materials

The supporting information can be downloaded at: https://www.mdpi.com/article/10.3390/ijms241411866/s1.
