# Supplementary material for: Engineering of Shikimate Pathway and Terminal Branch for Efficient Production of L-Tryptophan in Escherichia coli
Source: Int J Mol Sci. 2023 Jul 24;24(14):11866. doi: 10.3390/ijms241411866 (PMC10380740; doi:10.3390/ijms241411866)
Supplement: Supplementary file 1 [file ijms-24-11866-s001.zip › ijms-2477340-SI.pdf]

International Journal of Molecular Sciences

Engineering of Shikimate pathway and Terminal Branch for Efficient Production of L-tryptophan in *Escherichia coli*

Shuai Liu, Bing-Bing wang, Jian-Zhong Xu*, Wei-Guo Zhang*

The Key Laboratory of Industrial Biotechnology, Ministry of Education, School of Biotechnology, Jiangnan University, 1800# Lihu Road, Wuxi 214122, China

*Correspondence: xujianzhong@jiangnan.edu.cn (J.-Z.X.); zhangwg@jiangnan.edu.cn (W.-G.Z.); Tel./Fax: +86-510-85329312 (W.-G.Z.)

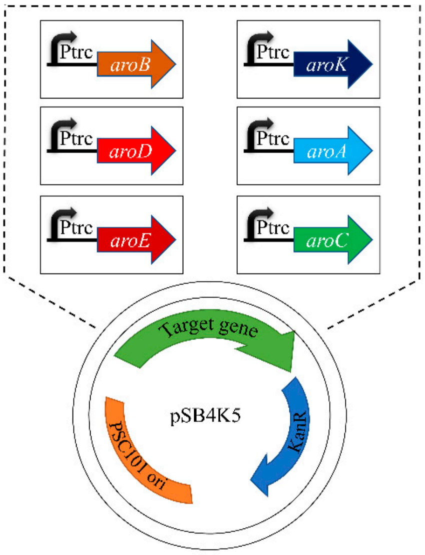

Figure S1. Schematic diagram of constructing plasmids (pSB4K5-aroB, pSB4K5-aroD, pSB4K5-aroE, pSB4K5-aroK, pSB4K5-aroA, and pSB4K5-aroC) for overexpressing shikimate pathway genes

Table S1 Gradient elution program of HPLC for detection of L-trp, L-tyr, L-phe, and anthranilate.

Time（min）	methanol (A)	0.05% H_3PO_4 (B)
0-3	2%	98%
3-22	2-80%	98-20%
22-25	2-80%	98-20%
25-26	80–2%	20-98%

an Inertsil ODS-SP column (250 mm × 4.6 mm i.d., 5 um, GL Sciences, Japan) was used for gradient elution. The flow rate was set to 1 ml/min, and the detection wavelength was 210 nm.

The base sequence of pSB4K5 is as follows :

cgaagaaaggcccacccgtgaaggtgagccagtgagttgattgctacgtaattagttagttagcccttagtgact
cgaattcgcggccgcttctagagcacaggccacagtaatacgactcactatagggttgtcatgtgtatgttgggg
gatcccgactggcgagagccaggtaacgaatggatcccccacatactttgttgatcccgcaggattcggctcgt
gtagctcattagctccgagccgagtcctcgaatacgagctgggcacagaagatatggcttcgtgcccaggagg
tgttcgcacttctctcgtgttcgattgtgggatcaatcatggcaaatgcggccgccgaccagaatcatgcaagtgc
gtaagatagtcgcgggtcggtggtcgcattacttagcataaccccgcggggcctcttcgggggtctcgcggggt
tttttgctgaaacggctgctaacaaagcccgaaaggaagctgagttggctgctgccaccgctgagcaataacta
gcataaccccttggggcctctaaacgggtcttgaggggttttttgctgaaaggaggaactacactgtctgcagga
gtcactaagggttagttagttagattagcagaaagtcaaaagcctccgaccggaggcttttgactaaaacttccct
tggggttatcattggggctcactcaaaggcggtaatcagataaaaaaaatccttagctttcgctaaggatgatttc

tgctagtattattagaaaaactcatcgagcatcaaatgaaactgcaatttattcatatcaggattatcaataccatat
ttttgaaaaagccgtttctgtaatgaaggagaaaactcaccgaggcagttccaaagaatggcaaggtcctggta
acggtctgcgattccgacccgtccaacatcaatacaacctattaatttcccctcgtcaaaaataaggttatcaagtg
agaaatcaccatgagtgacgactgaatccggtgagaatggcaagagcttgtgcatttctttccagacttgttcaa
caggccagccattacgctcgtcatcaaaatcactcgcatcaaccaaaccgttattcatgcgtgattgcgcctgagc
aagacgaaatacacgatcgctgttaaaaggacaattacaaacaggaatcgaatgtaaccggcgcaggaacac
ggccagcgcatcaacaatattttcacctgaatcaggatattcttctaatacctggaaggctgttttcccaggaatcg
cggtggtgagtaaccacgcatcatcaggagtacggataaaatgcttgatggtcgggagaggcataaactccgt
cagccagttgagacggaccatctcatctgtaacatcattggcaacgctacctttgccatgtttcagaaacaactctg
gcgcatcgggcttcccatacaagcgatagattgtcgcacctgattgcccgacattatcgcgagcccatttataccc
atataaatcagcgtccatgttggagtttaagcgcggacgggagcaagacgtttcccgttgaatatggctcataac
accccttgtattactgtttatgtaagcagacagttttattgttcatgatgatatatttttatcttgtgcaatgtaacatca
gagattttgagacacaacgtggctttgttgaataaatcgaacttttgctgagttgaaggatcagctctagtagttac
attgtcgatctgttcatggtgaacagctttgaatgcaccaaaaactcgtaaaagctctgatgtatctatctttttaca
ccgttttcatctgtgcatatggacagttttccctttgatatgtaacggtgaacagttgttctacttttgtttgttagtctt
gatgcttcactgatagatacaagagccataagaacctcagatccttccgtatttagccagtatgttctctagtgtgg
ttcgttgtttttgcgtgagccatgagaacgaaccattgagatcatacttactttgcatgtcactcaaaaattttgcctc
aaaactggtgagctgaatttttgcagttaaagcatcgtgtagtgtttttcttagtccgttatgtaggtaggaatctga
tgtaatggttgttggtattttgtcaccattcatttttatctggttgttctcaagttcggttacgagatccatttgtctatct
agttcaacttggaaaatcaacgtatcagtcgggcggcctcgcttatcaaccaccaatttcatattgctgtaagtgtt
taaatctttacttattggtttcaaaacccattggttaagccttttaaactcatggtagttattttcaagcattaacatga
acttaaattcatcaaggctaatctctatatttgccttgtgagttttcttttgtgttagttcttttaataaccactcataaat
cctcatagagtatttgttttcaaaagacttaacatgttccagattatattttatgaatttttttaactggaaaagataag
gcaatatctcttcactaaaaactaattctaatttttcgcttgagaacttggcatagtttgtccactggaaaatcccaaa
gcctttaaccaaaggattcctgatttccacagttctcgtcatcagctctctggttgctttagctaatacaccataagca
ttttccctactgatgttcatcatctgagcgtattggttataagtgaacgataccgtccgttctttccttgtagggttttc
aatcgtggggttgagtagtgccacacagcataaaattagcttggtttcatgctccgttaagtcatagcgactaatc
gctagttcatttgctttgaaaacaactaattcagacatacatctcaattggtctaggtgattttaatcactataccaat
tgagatgggctagtcaatgataattacatgtccttttcctttgagttgtgggtatctgtaaattctgctagacctttgc
tggaaaacttgtaaattctgctagaccctctgtaaattccgctagacctttgtgtgttttttttgtttatattcaagtggt
tataatttatagaataaagaaagaataaaaaaagataaaaagaatagatcccagccctgtgtataactcactact
ttagtcagttccgcagtattacaaaaggatgtcgcaaacgctgtttgctcctctacaaaacagaccttaaaaccta
aaggcttaagtagcaccctcgcaagctcgggcaaatcgctgaatattccttttgtctccgaccatcaggcacctga
gtcgctgtctttttcgtgacattcagttcgctgcgctcacggctctggcagtgaatgggggtaaatggcactacag
gcgccttttatggattcatgcaaggaaactacccataatacaagaaaagcccgtcacgggcttctcagggcgtttt
atggcgggtctgctatgtggtgctatctgactttttgctgttcagcagttcctgccctctgattttccagtctgaccac
ttcggattatcccgtgacaggtcattcagactggctaatgcacccagtaaggcagcggtatcatcaacaggctta
cccgtcttactgtccctagtgcttggattctcaccaataaaaaacgcccggcggcaaccgagcgttctgaacaaat
ccagatggagttctgaggtcattactggatctatcaacaggagtccaagcgagctcgtaaacttggtctgacagc
tctagctccggcaaaaaaacgggcaaggtgtcaccaccctgccctttttctttaaaaccgaaaagattacttcgcg
tttgccacctgacgtctaagaaaaggaatattcagcaatttgcccgtgc
